# Heatwave sears China: Need for actionable climate change adaptation to protect public health

**DOI:** 10.1016/j.lanwpc.2022.100568

**Published:** 2022-08-10

**Authors:** John S. Ji

**Affiliations:** Vanke School of Public Health, Tsinghua University, Beijing, China

Adaptation refers to adapting life to a changing climate to reduce our risk from the harmful impact. Throughout history, societies and populations adapted to changing climate with varying degrees of success. While climate change is a global issue, the extreme weather events are felt on a local scale. This past month, a series of intense heat waves engulfed dozens of cities across China with as many as 900 million people experiencing high temperatures. A total of 71 weather stations reported the hottest weather ever on record. Eighteen provincial-level regions experienced temperatures exceeding 35 degrees Celsius, and several places recorded temperatures above 44 degrees Celsius. This smelting heatwave prompted the country's national meteorological observatory to issue the “orange alert” temperature warning on July 14th, 2022.^1^

Public health scientists, policymakers, and the public are keenly aware of the health hazard of extreme temperatures. To measure the impact of non-optimal temperatures, researchers developed lageffect models used for time series analyses, since health outcomes onset may not occur on the same day. Pooled relative risks estimates from epidemiology studies, satellite-derived and ground monitors of temperatures, and exposure-response curves are then used as input parameters to calculate the disease burden for the general population. If estimates are correct, exposure to non-optimal temperatures is currently one of China's highest causes of death. In the July 2022 issue of *The Lancet Regional Health - Western Pacific*, research on mortality burdens due to temperature estimated 593·9 thousand excess deaths that were attributable to non-optimal temperatures in a single year in China in 2019 (death rate 41.8 per 100,000). High temperature contributes to 13.9 thousand deaths (1.0 per 100,000) and low temperature contributes to a staggering 580.8 thousand deaths (40.8 per 100,000). Like all insults to the body, those with existing non-communicable diseases tend to be the most vulnerable. Geographically, Tibet has the highest cold-spell deaths, and Hainan has the highest heat-wave deaths.^2^ Many more extreme temperature events are bound to occur in the future, with even more aberrant timing of onset and duration due to changing climate patterns. In addition to the effects on health (heat stroke and mortality), the immediate impacts will be peak electricity overload on the grid system due to greater energy demand, evapotranspiration, crop droughts, and altering of biodiversity and ecological balance.

China has one of the fastest urbanization rates, and further metropolis integration of megacities is underway, such as Beijing-Tianjin-Hebei, Yangtze River Delta (Anhui, Jiangsu, Shanghai, Zhejiang), and Pearl River Delta (Guangdong, Hong Kong, Macau). Nearly half a billion people live and work in these clusters. The sprawl of concrete in cities to make room for more human settlement erected a man-made built environment with impervious surfaces through the peri-urbanization process. This has inadvertently generated the urban heat island effect, marked by heightened air, and ground temperatures. Urban density is not entirely undesirable, however, as it could have efficiency gains through higher productivity and reduction in energy use as economic co-benefits. Thus, could cities have an advantage in climate change adaptation, and what more can be done? The 2021 report of the Lancet Countdown on health and climate change keeps track of several indicators for adaptation delivery and implementation: emergency preparedness, air conditioning, and urban greenspace.^3^ With carbon neutrality targets set, policymakers in China submitted renewed its nationally determined contribution (NDC) to the United Nations Paris Agreement and laid out plans for proactive adaptation through climate-resilient city pilot in 28 locations.^4^ Progress and successes are still up in the air; however, changes are underway with international cooperation on early warning and preparedness for climate disasters, conservation and rerouting of water resources, increased forestry for carbon sink with health co-benefits for cooling, and infrastructure projects to protect coastlines. While we await global cooperation on climate change targets, individuals and local communities should also make our own adaptation plans, and be ready for the next heat- and cold-wave shocks.

## Declaration of interests

None Declared.
